# The Effect of Probiotics on the Production of Short-Chain Fatty Acids by Human Intestinal Microbiome

**DOI:** 10.3390/nu12041107

**Published:** 2020-04-16

**Authors:** Paulina Markowiak-Kopeć, Katarzyna Śliżewska

**Affiliations:** Institute of Fermentation Technology and Microbiology, Department of Biotechnology and Food Sciences, Lodz University of Technology, 90-924 Łódź, Poland

**Keywords:** probiotics, human health, SCFA, intestinal microbiome, metabolites of bacteria

## Abstract

The relationship between diet and the diversity and function of the intestinal microbiome and its importance for human health is currently the subject of many studies. The type and proportion of microorganisms found in the intestines can determine the energy balance of the host. Intestinal microorganisms perform many important functions, one of which is participation in metabolic processes, e.g., in the production of short-chain fatty acids—SCFAs (also called volatile fatty acids). These acids represent the main carbon flow from the diet to the host microbiome. Maintaining intestinal balance is necessary to maintain the host’s normal health and prevent many diseases. The results of many studies confirm the beneficial effect of probiotic microorganisms on the balance of the intestinal microbiome and produced metabolites, including SCFAs. The aim of this review is to summarize what is known on the effects of probiotics on the production of short-chain fatty acids by gut microbes. In addition, the mechanism of formation and properties of these metabolites is discussed and verified test results confirming the effectiveness of probiotics in human nutrition by modulating SCFAs production by intestinal microbiome is presented.

## 1. Introduction

The most attention in research on human microbiome is devoted to the analysis of the diversity of microorganisms present in the digestive system, especially in the intestines. This metagenome is often called the third main mammalian genome, in addition to the nuclear and mitochondrial genomes. Nutrient absorption and energy regulation depend on environmental and lifestyle factors (dietary habits, drug treatments, intestinal motility and stool frequency and consistency) as well as the bacteria commonly found in the gastrointestinal tract (referred to as the intestinal or gut microbiome). Among the about 60 phyla of bacteria currently known, only some are present in human intestines (e.g., *Firmicutes*, *Bacteroides*, *Actinobacteria*, *Fusobacteria*, *Proteobacteria*, *Verrucomicrobia*, *Cyanobacteria*, and *Spirochaetes*) [1]. Two bacterial phyla, Gram-positive *Firmicutes* (*Lactobacillus* spp., *Bacillus* spp., and *Clostridium* spp.) and Gram-negative *Bacteroidetes*, predominate in human gut [2].

The type and proportions of microorganisms found in the intestines, i.e., the enterotype may determine the host’s energy balance. In addition, the intestinal microbiome supports the biotransformation of numerous chemical compounds. Due to their metabolic abilities, intestinal microorganisms enable the transformation of complex nutrients, such as plant cell wall components (cellulose, pectin, hemicellulose, lignin) and mucins into simple sugars that are fermented to form short-chain fatty acids (SCFAs, mainly acetate, propionate and butyrate) [1]. The intestinal microbiome also plays an important role in modulating mucosal homeostasis of immune cell subpopulations and the synthesis of certain vitamins [3,4,5,6]. The activity of microorganisms in the intestines has a significant effect on the action of gastrointestinal hormones, maintaining intestinal homeostasis, regulates the proliferation and differentiation of epithelial cells, and prevents mucosal colonization by pathogenic microorganisms [7,8,9].

An imbalance of the intestinal microbiome and a decrease in the number of bacteria producing metabolites such as SCFAs (e.g., acetic, propionic and butyric acid) often occur in patients with inflammatory bowel diseases (IBD), irritable bowel syndrome (IBS), type 2 diabetes (T2D), obesity, autoimmune disorders or in cancer patients [10,11,12,13,14]. A lower abundance of specific bacteria and SCFAs leading to gut barrier dysfunction, low-grade inflammation and altered glucose, lipid and energy homeostasis are characteristic for obesity and T2D [15]. The composition of the intestinal microbiota was found to be different in people with obesity than in people of normal weight [16]. This is confirmed, among others, by studies in which *Faecalibacterium prausnitzii* was found to be the most abundant (about 5% of the bacterial population) in the intestines of healthy adults, while overweight people had a higher number of *Firmicutes* and *Actinobacteria* and a lower number of *Bacteroidetes*, *Verrucomicrobia* and *F. prausnitzii* [17]. Thus, research results indicate that obesity is associated with a decrease in the number of *Bacteroidetes* and an increase in the number of *Firmicutes*, with the intestinal microbiome of an obese person being less diverse than that of a lean person [18,19]. It should also be noted that *Faecalibacterium prausnitzii* is the first anti-inflammatory commensal bacterium identified on the basis of human clinical data and is also one of the major butyrate-producer of the human intestinal microbiome [20,21]. Other studies have shown a higher concentration of SCFAs (especially butyric and propionic acid) in the feces of overweight children compared to healthy children [2]. Similar results were obtained in a study of Swiss children, in this case the concentration of butyric and propionic acid was also significantly higher in the feces of overweight children [22]. However, different results were obtained for overweight Japanese and Mexican children, where SCFAs concentration was higher in the feces of children with normal weight [23,24]. Therefore, the trend of SCFAs content in feces cannot be dependent on a specific BMI group. Instead, the concentration of these acids is more associated with dysbiosis of the intestinal microbiome, genetics, environmental factors and diet [2].

There are many methods for modulating the intestinal microbiome. One of these is the use of probiotics, which can be helpful in maintaining or restoring homeostasis in the intestines to improve human health and prevent many diseases. According to the definition formulated in 2002 by the FAO (Food and Agriculture Organization of the United Nations, Rome, Italy) and the WHO (World Health Organization, Geneva, Switzerland), probiotics are live microorganisms which, when administered in adequate amounts, confer a health benefit on the host [25]. The definition was maintained by the International Scientific Association for Probiotics and Prebiotics (ISAPP) in 2013 and is still currently used [26]. The most commonly used probiotics are lactic acid bacteria (LAB) and bifidobacteria [8]. The growth and metabolic activity of probiotic microorganisms can be selectively stimulated by various types of carbohydrates that are not digested by the host (prebiotics) [9]. The combinations of probiotics with prebiotics (called synbiotics) are able to shift the predominant bacteria and the production of SCFAs of fecal microorganisms in a model system of the human colon [27].

## 2. Short-Chain Fatty Acids

Organic acids, principally the short-chain fatty acids (SCFAs) are formed in the GI tract in millimolar quantities and especially occur in high amounts in those areas where anaerobic microorganisms are predominant. SCFAs are volatile saturated fatty acids that have in their chain 1-6 carbon atoms in the aliphatic chain, existing in a straight or branched conformation [28]. In this review, attention has been focused on SCFAs with a simple conformation, which include formic, acetic, propionic, butyric, valerian, and caproic acids (Table 1).

SCFAs represent the main carbon flow from the diet to the host microbiome [30]. The formation of these acids is relatively well-known and described [31,32]. The concentration and ratio of resulting SCFAs depend not only on the composition of the microbiome and the number of individual microorganisms in the colon, but also on the type of dietary fibers supplied to the microorganisms as a substrate in the fermentation process, and thus on the diet [8]. The most common are acetic acid, propionic acid and butyric acid (in a 3:1:1 molar ratio), which constitute 90%–95% of SCFAs present in the human colon, while a smaller proportion of these is formic acid [28].

In addition, the fermentation of selected, often rapidly fermentable non-digestible carbohydrates (NDCs) produces another organic acid-lactic acid [30]. Although it does not belong to the group of SCFAs, this acid can be produced by lactic acid bacteria, e.g., the genera *Lactobacillus* and *Bifidobacterium* [32]. However, under normal conditions it is not accumulated in the colon due to the presence of some bacterial species, e.g., *Eubacterium hallii*, that can convert lactate into different SCFAs [32]. Metagenomic analyses have greatly facilitated the identification of the types of bacteria responsible for the production of SCFAs and lactic acid (Table 2).

### 2.1. Bacterial Fermentation Involved into Production of SCFAs

Endogenous short-chain fatty acids are formed by bacterial fermentation of food fiber and NDCs, which become available to intestinal microorganisms in the large intestine. In addition to resistant starch (RS), plant-derived NDCs include non-starch polysaccharides (NSP), oligosaccharides (prebiotics), oligofructose, disaccharides (lactose, stachyose, raffinose) and monosaccharides e.g., alcohols (sorbitol, mannitol) [7]. There are four types of resistant starch (RS1–RS4) present in the human diet that are resistant to degradation in the small intestine [44,45]. The type of RS has a significant effect on the composition of the intestinal microbiome [46]. In the case of oligosaccharides, particularly important are prebiotics defined as a nonviable food component that confers a health benefit on the host associated with modulation of the microbiota [47]. However, endogenous indigestible carbohydrates include mucin and milk oligosaccharides [7].

Fermentation is an anaerobic redox process in the cytoplasm in which organic compounds are both electron donors and acceptors. In the fermentation process, electrons detached from the oxidized substrate are transferred by NADH directly to the endogenous acceptor. ATP is formed as a result of substrate phosphorylation, with the participation of the corresponding phosphoglycerate, pyruvate, acetate or butyrate kinases. During carbohydrate fermentation, the final electron acceptor is pyruvate or the compounds that are produced from it. The end products of fermentation are various short chain carboxylic acids (e.g., formic, acetic, lactic, butyric, propionic), CO_2_, H_2_, ethanol, glycerol, acetoin, 2,3-butanediol. Importantly, bacterial growth in populations mixed with other microorganisms can affect the type and amount of products produced during fermentation. The substrates most commonly used by microorganisms in the fermentation process are hexoses and pentoses [1].

**Figure 1:**
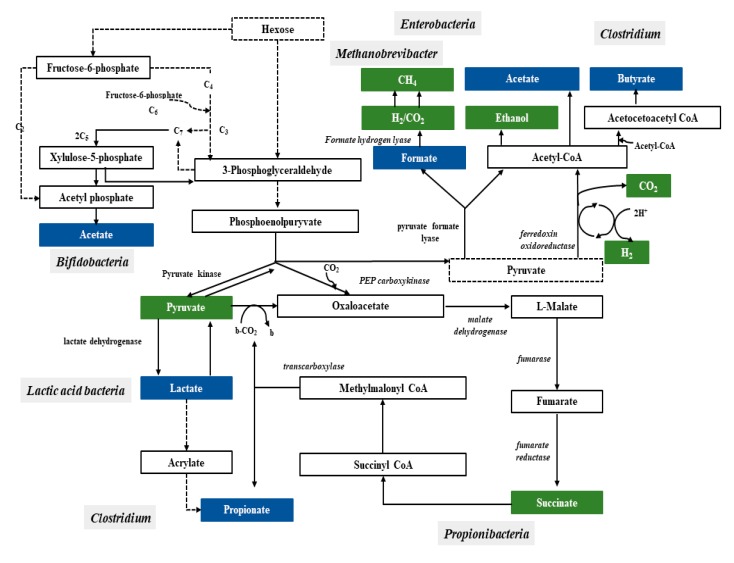
Pathways leading to SCFAs and lactic acid production by intestinal bacteria [48].

Bacteria have a variety of pathways to transform sugars. These sugars are first phosphorylated and then in glycolysis (Embden–Meyerhof–Parnassian pathway), in the Entner–Doudoroff pathway or in the *Bifidobacterium* pathway are transformed into pyruvate or into pyruvate and additional acetyl-phosphate. The Embden–Meyerhof–Parnassian pathway, the major colonic pathway for the catabolism of hexoses, occurs in enterobacteria, clostridia, homofermentative lactic acid bacteria, and propionibacteria, and produces only pyruvate as a partial oxidation product) [48]. The Entner–Doudoroff pathway is used in the fermentation metabolism by e.g., *Zymomonas* (alcoholic fermentation), as well as *Escherichia coli* in gluconate fermentation. The *Bifidobacterium* pathway is active in bacteria of the genus *Bifidobacterium*, inhabiting, among others, the human digestive system. Two acetate molecules and one lactate are formed in this pathway. In the phosphoketolase pathway that occurs in heterofermentative lactic acid bacteria or the *Bifidobacterium* pathway, an additional molecule of acetyl-phosphate is generated (Figure 1) [49].

In a mixed population such as the intestinal microbiome, carbohydrate breakdown into a mixture of acids involves more than one species. This type of fermentation is called mixed acid fermentation or *Enterobacteriaceae* fermentation and is carried out by some bacteria belonging to this family, including *Escherichia*, *Proteus*, *Salmonella*, and *Shigella* [1]. The fermentation products of some species are substrates for fermentation or incorporated as intermediate metabolites into the metabolic pathways of other species, resulting in substrates being sequentially fermented. Lactate, ethanol, and pyruvate are diminished by subsequent bacterial utilization and SCFAs production. Accordingly, the main final products of sugar catabolism are SCFAs, acetate, propionate and butyrate that account for 85%–95% of total SCFAs in all regions of the colon. Other fermentation end products, such as caproate and valerate, occur in lower amounts [48].

Acetic acid is the most abundant SCFAs in the colon, accounting for more than half of the total SCFAs found in feces [49]. Intestinal microorganisms can produce acetic acid through two major metabolic processes. Most often it is fermentation of indigestible carbohydrates, while about 1/3 of acetic acid is formed as a result of synthesis from hydrogen and carbon dioxide or formic acid by acetogenic bacteria through the Wood–Ljungdahl pathway [31,35].

Some types of *Clostridium* (*C. acetobutylicum*, *C. butyricum*, *C. pasteurianum*, *C. perfringens*) participate in butyric fermentation, as well as e.g., *Butyrivibrio fibrisolvens* and *Fusobacterium nucleatum*. The end products are butyric acid, a small amount of acetic acid and CO_2_ and H_2_. Some species may also form lactic acid and/or ethanol as well.

For propionic fermentation, the main substrates are glucose and lactate. Its course varies depending on the bacteria; it can occur that it forms succinate or acrylate [1].

Bacteria are capable of fermenting sugar degradation products (glycerol, citrate, malate, succinate, pyruvate, lactate, ethanol, acetate), and a small share of dietary protein fermentation processes in the production of SCFAs has been shown, mainly in the form of acetic and propionic acid [50]. Bacteria of the genus *Clostridium* are capable of fermenting amino acids. In this process, carbon dioxide, hydrogen, acetate, as well as ammonia and butyrate may form, which have an unpleasant odor. In addition, amino acids such as valine, leucine and isoleucine resulting from the anaerobic breakdown of proteins can be converted into compounds with strong odor, such as isobutyric, isovaleric and hexanoic acids, as well as cadaverine, putrescine, other amines, and hydrogen sulfide and methylmercaptan [1]. Excessive accumulation of isobutyric acid and isovaleric acid indicates a malfunctioning fermentation and digestion processes. These are putrefactive acids, the increased production of which may be associated with an excess of unabsorbed amino acids or proteins reaching the intestines. The possibility of blood in the intestinal contents and the overly intensive development of pathogenic microbiota in the small intestine should also be taken into account, where access to protein compounds is facilitated [51].

### 2.2. Functions of Short-Chain Fatty Acids

SCFAs have been shown to have a very positive effect on the energy metabolism of mammals that use them together with glucose as a metabolic fuel [52]. It has been estimated that the use of SCFAs as an energy source can provide up to 10% of the host’s daily calories [53]. The presence of these acids in the human body, mainly acetic, butyric and propionic acids in sufficient quantities is essential for the health and well-being of the host [5]. However, the production of these acids requires the presence of appropriate substrates (dietary fiber and prebiotics) needed for the proper course of the fermentation processes.

**Figure 2:**
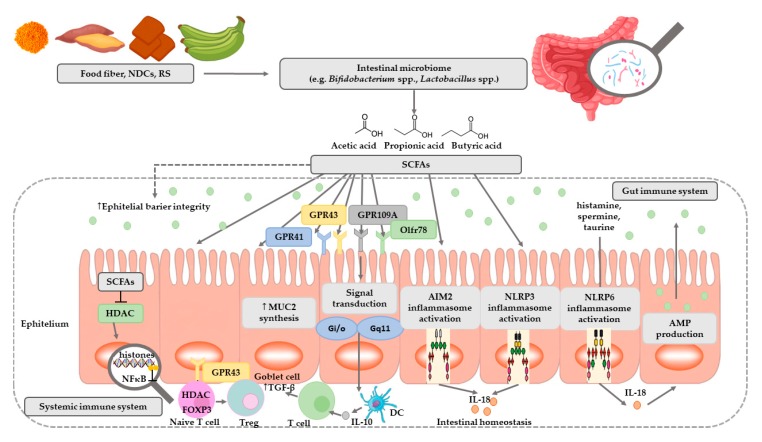
The role of SCFAs in regulation of intestinal homeostasis. SCFAs (acetic, propionic, and butyric acid) are produced by intestinal microbiome in fermentation of undigested food fiber, non-digestible carbohydrates (NDCs) or resistant starch (RS). SCFAs are as energy substrates for colonocytes and regulate intestinal barrier function (synthesis of mucin-MUC2) and immune system through G-protein-coupled receptors (GPR41, GPR43, GPR109A) and Olfr78 receptor signaling. SCFAs regulate the histone deacetylase (HDAC) activity which affects inhibition of nuclear factors (nuclear factor-κB; NF-κB). SCFAs affect the differentiation of regulatory T (Treg) cells and the production of interleukin-10 (IL-10) with the participation of GPR43. SCFAs also regulate dendritic cell (DC) function. In addition, SCFAs influence AIM2 and NLRP3 inflammasomes activation which then affects production of interleukin-18 (IL-18) and enhanced epithelial barrier function. Moreover, NLRP6 inflammasome activation and secretion of IL-18 regulate the production of intestinal antimicrobial peptides (AMPs) [54,55]. Abbreviations: FOXP3-forkhead box P3; TGF-β-transforming growth factor β.

SCFAs play a very important role in maintaining intestinal and immune homeostasis in the human body (Figure 2). 

SCFAs are speculated to have a mediational role in the microbiota–gut–brain axis crosstalk [56]. Two major SCFAs signaling mechanisms have been identified, namely inhibition of HDACs and activation of GPCRs—The binding partners of GPR41 and GPR43 (Table 3) [57,58].

SCFAs play a very important role in regulating pH, increasing the absorption of calcium, iron, as well as magnesium, and are beneficial for glucose and protein metabolism in the liver. In addition, these acids affect the maintenance of the normal structure, integrity and function of the intestines [7]. They show anti-inflammatory activity, which involves inhibiting the activity of inflammatory mediators in the intestinal epithelium, and thus inhibiting the activation of NFκB macrophages, which are the main source of cytokines in the course of the inflammatory process of inflammatory bowel diseases [7]. These acids are the primary source of energy for colonocytes [62,63]. It has been shown that the source of 70% of the energy used by intestinal epithelial cells (IEC) is butyric acid produced by commensal bacteria, especially such as *Ruminococcus* and *Faecalibacterium* (Table 4) [37]. In addition, by simulating the growth of saprophytic microflora, SCFAs inhibit the development of pathogenic microorganisms such as *Escherichia coli*, *Salmonella*, or *Campylobacter*, competing for colonization sites [8]. Studies have shown that butyric acid stimulates the expression of the MUC2 gene in cell lines and the production of mucin, and the sticky layer it creates protects the intestinal epithelium from contact with toxins and pathogenic microorganisms [64]. In contrast, studies of programmed cell death from a tumor line have demonstrated the effectiveness of butyric acid in inhibiting their development and inducing the process of apoptosis [65,66,67]. In addition, butyric acid and propionic, acetic, and valeric acids have been shown to induce apoptosis (Table 4) [68].

SCFAs increase the amount of mucus produced and the speed of blood flow. More importantly, they provide acetyl-CoA used in the process of fat biosynthesis and cell membrane production, guaranteeing the integrity of mucous membranes [69]. There are indications that SCFAs are key mediators of the beneficial effects of intestinal microbiota. SCFAs also directly modulate host health through a number of tissue-specific mechanisms associated with intestinal barrier function, glucose homeostasis, immunomodulation, appetite regulation, obesity, and have a direct and indirect effect on cardiovascular disease (CVD) risk markers [70].

At present, relatively little is known about the function of formic acid in the intestines. There are indications that its presence is associated with methanogenesis and its concentration may be elevated during inflammation (Table 4) [71,72]. Acetic acid concentration in the colon is the highest of all SCFAs, and in cells it is a key factor in the metabolism of carbohydrates and fats [73]. In addition, acetic acid is absorbed by the liver, where it participates in the synthesis of cholesterol (Table 4) [74]. Propionic acid is produced in the human gut mainly by *Bacteroidetes* and *Firmicutes* [75]. This acid is an inhibitor of gluconeogenesis and cholesterol synthesis in the liver [76]. In addition, it has antibacterial and anti-inflammatory effects, taking part in the protection of human intestines against pathogens [8,77]. Butyric acid exerts the strongest anti-inflammatory effect of all SCFAs [7]. The cause of the inflammatory process of the intestinal mucosa, which accompanies many pathological processes, is a lack of energy. Butyric acid is the main source of energy for intestinal epithelial cells. Butyric acid has a beneficial immunoregulatory effect on intestinal epithelial cells and other mucosal cell populations. It modulates gene expression by affecting both stimulants and inhibitors of expression. Some of these mechanisms are based on histone hyperacetylation due to inhibition of the histone deacetylase enzyme activity (Table 4) [78].

Unlike other SCFAs, the role of valeric acid in gut health is not fully understood. In a limited number of studies, it was found that valeric acid can stimulate the growth of intestinal epithelium and have a beneficial effect on the pathogenesis of diseases such as colitis, cardio-metabolic diseases and cancer (Table 4) [79,80,81].

## 3. The Effect of Probiotics on SCFAs Production by Intestinal Microbiome

In order to determine the effect of probiotics on the SCFA production to human intestinal microbiome of a literature review was conducted using the database Web of Science, Medline, Elsevier. To identify relevant studies, articles from 1996 to 2020 in databases were searched. The following keywords were used in the search: Probiotic, SCFA, colorectal cancer, obesity, diabetes, type 2 diabetes, atopic dermatitis, autism spectrum disorders, cardiovascular disease, gastrointestinal disorders, etc. The search was restricted to publications in English. A comprehensive full-text review of identified studies was conducted after the title and abstract screening of potentially relevant articles.

Intestinal microorganisms, due to their participation in metabolic processes, have a significant impact on the metabolism of the whole body. The balance of this microbiome is necessary to maintain the proper health of the host and prevent many diseases. Therefore, researchers hypothesized that in people in whom the number of certain groups of microorganisms is too low, deliberate reproduction or administration of these microorganisms may be beneficial [90].

In in vitro human intestinal model studies (the M-SHIME^®^ system), effect of an aqueous probiotic suspension (Symprove^TM^, containing *Lactobacillus acidophilus* NCIMB 30175, *Lactobacillus plantarum* NCIMB 30173, *Lactobacillus rhamnosus* NCIMB 30174, and *Enterococcus faecium* NCIMB 30176) on bacterial diversity was tested. SCFAs production and inflammatory markers after 3 weeks dosing of probiotic was found [91]. The results confirmed colonization and growth of three probiotic species in the luminal and mucosal compartments of the proximal and distal colon, and growth of a last species in the luminal proximal colon. The colonization and growth probiotic bacteria leaded to higher proximal and distal colonic lactate concentrations. In effect, the lactate stimulated growth of lactate-consuming bacteria and resulting in increased SCFAs production, especially butyrate. Additionally, an immunomodulatory effect of the probiotics was seen; production of anti-inflammatory cytokines (IL-10 and IL-6) was increased and production of inflammatory chemokines (IL-8, CXCL 10 and MCP-1 and) was reduced [91].

In another study, the effect of oral consumption of *Lactobacillus plantarum* P-8 (Lp-8) on human intestinal microflora, and SCFAs of different aged adults was tested [92]. 33 volunteers including young (mean age 26 years), middle-aged (mean age 51 years), and elderly (mean age 76 years) received Lp-8 (6 × 10^10^ colony forming units daily) for 4 weeks. The increase in *Bifidobacterium* and other beneficial bacteria was found, whereas *Desulfovibrio* and other opportunistic pathogens decreased after taking probiotic for 4 weeks. A statistically significant increase in acetate and propionate levels in all age groups was found, which reached a peak after 5 weeks in all age groups [92].

Other scientists also tested the anti-aging potential of a probiotic in combination containing *Lactobacillus paracasei* ssp. *paracasei* BCRC 12188, *Lactobacillus plantarum* BCRC 12251, and *Streptococcus thermophilus* BCRC13869. The studies used the murine model in vivo, wherein the aging induced d-galactose [93]. The 12-week study was conducted on 15 mice. It turns out that long-term administration of the probiotic mixture by increasing the production of SCFAs (might regulate antioxidant enzymes by inducing expression of Nrf2 or HO-1) and inhibiting cell apoptosis and brain injury, resulting in improved memory and learning abilities in d-galactose–treated aging mice [93].

Moreover, in different research the potential health benefits of a fermented salami with a probiotic *Lactobacillus rhamnosus* HN001 and added citrus fiber for 4 weeks in 24 health people was tested [94]. It was found that the inflammatory markers CRP and TNFα decreased significantly after intervention, suggesting a less inflammatory environment after reformulated salami consumption. In addition, antioxidant plasmatic markers also improved and butyrate production was significantly increased within the intervention group [94].

### 3.1. Colorectal Cancer (CRC)

CRC is the third most prevalent cause of death among the different types of cancer and the highest incidence being in developed countries [95]. It is estimated that by 2035, 24.4 million new cases of CRC will be diagnosed annually [96]. CRC is strongly correlated with decreased levels of SCFAs and microbiome dysbiosis [95]. Administration of *Butyrivibrio fibrisolvens* MDT-1, (known for their high production of butyrate) in mouse model of colon cancer, inhibited progression of tumor development, affecting also the reduction of β-glucuronidase and increasing the immune response [97]. Currently, it is suggested to modulate SCFA-producing bacteria through dietary intervention with fermentable fibers as a possible treatment for CRC [98]. In many in vitro studies have attempted to determine effects and potential mechanisms of action of probiotics in the inhibition of cancer cell proliferation. In the research on human colonic cancer cell line Caco-2, *Pediococcus pentosaceus* FP3, *Lactobacillus salivarius* FP25 and FP35, and *Enterococcus faecium* FP51 in different concentrations were tested [99]. Tested probiotics reduced cell proliferation. Mechanisms responsible for this effect were adhesion of probiotic bacteria to colon cancer cells and an increase in bioproduction of SCFAs [99]. On the other hand, in the study on human colonic cancer cells lines HT-29 and Caco-2 with using skimmed milk kefir and ayran, antioxidant and SCFAs activities were recognized as mechanisms responsible for the beneficial effect of the probiotic [100]. In this case, beneficial effect of probiotics was associated with lowering genotoxicity of fecal water added to the medium [100]. Other studies also confirmed the beneficial effect of SCFAs. In the research on human colonic cancer cells lines HCT116, SW1116, and Caco-2, the effect and the mechanism of action *Clostridium butyricum* ATCC 23857 and *Bacillus subtilis* ATCC 19398 were analyzed [101]. A beneficial effect was the reduction of cell proliferation and expression of inflammatory genes as a result of the presence of bacitracin or butyrate in the conditioned medium induced cell cycle arrest and apoptosis activation [101]. In the studies on male rats F344 (5 weeks old) the effect of *Lactobacillus salivarius* was tested [102]. As a result of the decrease in azoreductase activity and the intestinal population of *Bacillus* and *Ruminococcaceae* while increasing the number of intestinal populations of *Prevotella*, *Bacteroides*, *Lachnospiraceae*, *Clostridium*, and the concentration of SCFAs in feces, a decrease in aberrant crypt foci (ACF) incidence was found [102]. As part of the randomized, double-blind, placebo-controlled research 10 colorectal cancer patients and 20 healthy subjects were receiving *Lactobacillus gasseri* OLL271 6: LG21 for 12 weeks [103]. The effect of the tested probiotic was increase number of *Lactobacillus* spp. and decrease number of *Clostridium perfringens* in intestinal population. Moreover, increase in concentration isobutyric acid in feces and natural killer (NK) cell activity were found. In addition, supplementation of probiotic caused a decrease in pH and the synthesis of fecal putrefaction products (Table 5) [103].

In another research, effect of *Bifidobacterium lactis* LAFTI B94 administration in 17 healthy subjects (aged 45 to 75 years) for 4 weeks was tested [104]. The treatment resulted increase number of *Bifidobacterium lactis* in intestinal population, but changes in the pH, the SFCA fecal concentration, the serum hs-CRP and cytokines and also the crypt proliferation and cell height weren’t found (Table 5) [104].

### 3.2. Obesity

Obesity is a risk factor for CVD, dyslipidemia, hepatobiliary disease diabetes, premature death, and several cancers. There is an estimate of 1.7 billion people in the world that are overweight [109]. Individuals with obesity usually have an altered composition of intestinal microbiome, which suggest that intestinal microbiome can be considered the factor that creates the development of obesity [110]. Changes in the composition of the intestinal microbiome can be associated with obesity through changes in the form of reduced activity of the fasting-induced adipose factor (FIAF) and AMP-activated protein kinase (AMK), reduced production of SCFAs, increased inflammation or altered LPS-endocannabinoid (eCB) system regulators loops, and bile acid metabolism [110]. The host’s diet is considered to be the cause of these changes [109]. Manipulating microbial populations with probiotics in the presence of proper diets can reduce enteritis, improve intestinal barrier integrity, and increase the number of beneficial bacteria, which leads to weight loss [111]. Improvement of intestinal dysbiosis and obesity in animals and humans have been reported as a result of the use of probiotics [112,113]. However, animal studies still prevail, while there is little research on the effects of probiotics on SCFAs content in human feces. The administration of the probiotic strain *Lactobacillus acidophilus* DDS-1 has been shown to modulate the intestinal microbiome and the associated metabolic phenotype in aging mice. Probiotic administration increased the number of beneficial bacteria such as *Akkermansia* spp. and *Lactobacillus* spp and also caused an increase in butyric acid concentration, while reducing the production of inflammatory cytokines in serum and colonic explants [114]. The results of many other animal studies indicate that supplementation with probiotic *Lactobacillus* spp. bacteria induced SCFAs production by modulation of the intestinal microbiome [14,84,115,116]. For example, the probiotic bacteria *Bifidobacterium pseudocatenulatum* CECT 7765 administered to rats that received high-fat diets had an effect on increasing the intestinal barrier function, reducing endotoxemia and accelerating metabolism [117,118]. Satisfactory results for *Lactobacillus plantarum* strain no. 14 on improving metabolism have been shown in studies on obesity in animal models [119]. Other studies tested the efficacy of probiotic VSL#3 in preventing and treating obesity and diabetes in several mouse models [120]. It was confirmed that probiotic formula VSL#3 suppressed body weight gain and insulin resistance via modulation of the gut flora composition. In addition, it was found that VSL#3 promoted the release of the hormone GLP-1, resulting in reduced food intake and improved glucose tolerance. The VSL#3-induced changes were associated with an increase in the levels of a short-chain fatty acid (SCFAs), butyrate [117]. In another study feces from 40 Malaysian school-age children (19 of normal weight and 21 overweight children) who consumed a probiotic drink containing the *Lactobacillus casei* Shirota strain in two stages (four weeks each) were examined [2]. It was found that the consumption of the probiotic drink caused a significant increase in the number of *Lactobacillus* spp. and *Bifidobacterium* spp. in the composition of intestinal microbiota of overweight children. Both in normal weight and overweight children after four weeks of supplementation, a significant increase in SCFAs concentration, especially of propionic acid, was observed. In addition, a higher SCFAs concentration (especially butyric and propionic acid) was observed in the stool of overweight children than in normal weight children (Table 5) [2]. Also, when *Lactobacillus casei* Shirota was administered to children with obesity for six months, there was an improvement in the profile of intestinal microbiota and an increase in the concentration of acetic acid in the faces of children with obesity (Table 5) [24].

### 3.3. Type 2 Diabetes (T2D)

Diabetes may be closely associated with a higher risk of CVD. This is due to the compensatory effect leading to hyperinsulinemia and ultimately to various metabolic abnormalities. People with diabetes are also characterized by an altered intestinal microbiome, which can cause obesity, metabolic endotoxemia, B-cell dysfunction, systemic inflammation, and oxidative stress related to disease [109]. As a result of intestinal microbiome imbalance in T2D, production of SCFAs is reduced [90]. Probiotic therapy due to the increased production of SCFAs by intestinal bacteria as well as other functions may be effective in the treatment of diabetes.

In the study on 60 mice for 6 weeks, the anti-diabetic mechanisms of probiotics composed of 14 strains (Lactobacillus plantarum, Lactobacillus helveticus, Lactococcus lactis, Lactobacillus pentosus, Lactobacillus paracasei, Lactobacillus paracasei sbusp.tolerans, Lactobacillus mucosae, Lactobacillus rhamnosus, Lactobacillus harbinensis and Lactobacillus hilgardii, Issatchenkia orientalis, Candida ethanolica, Kluyveromyces marxianus, Pichia membranifaciens) isolated from traditional fermented camel milk was tested [121]. It was found that tested probiotics improve blood glucose and blood lipid parameters, which can lead to delayed development of T2D. In addition, these probiotics improve the function of the intestinal microbiome by increasing the levels of SCFA-producing bacteria (including lactic acid bacteria, Bifidobacterium, Clostridium leptum, Roseburia) and SCFAs (propionic acid and butyric acid) as well as the expression of cluaudin-1 and mucin -2, and decreasing Escherichia coli and lipopolysaccharide levels. The results obtained in these studies indicated that 14 composite probiotics might be considered to be a potential treatment method for treating patients with T2D [121]. 

The aim of other studies was to determine the effect of probiotic (fermented goat’s milk containing *Lactobacillus acidophilus* La-5 and *Bifidobacterium animalis* subsp. *lactis* BB-12) on glycemic control, lipid profile, inflammation, oxidative stress and SCFAs in T2D [105]. In a double-blind, randomized, placebo-controlled trial 50 volunteers consumed daily 120 g/d of fermented milk for 6 weeks. The control sample was the group receiving conventional fermented goat milk contained *Streptococcus thermophilus* TA-40. At end of trial, the proportion of propionic: acetic: butyric acids, taking into account the mean values, was also similar: 10:8:1 in the control group and 14:10:1 in the probiotic group. The authors found that administration of the test probiotic improves glycemic control in people with T2D, however, the intake of fermented goat’s milk seems to be involved with changes in inflammatory cytokines (TNF-α and resistin) and in the acetic acid concentrations (Table 5) [105].

### 3.4. Cardiovascular Disease (CVD)

CVD is the main cause of morbidity and mortality in the world, there is currently a search for methods of treating elevated blood cholesterol. Researchers suggest that high-fat diet consumption has been associated with gut dysbiosis and lead to dyslipidemia, hypertension and T2D mellitus [122]. In addition, SCFAs have the ability to modulate CVD risk factors, including blood pressure reduction and regulation of glucose and lipid homeostasis [123].

To this end, the impact of Daily Body Restore (DBR) (a proprietary blend of nine probiotic organisms of the genera *Lactobacillus* and *Bifidobacterium* and ten digestive enzymes) on cholesterol metabolism using an in vitro system and a mouse model of hypercholesterolemia induced by a high fat diet. Hypercholesterolemic mice were supplemented with DBR in their drinking water for eight weeks and compared to control mice given low fat diets or unsupplemented high fat diets. As a result, it was found that the probiotic-enzyme supplement used increased the microbiological production of propionic acid in colon reactors and also lowered harmful LDL and increased HDL levels in a mouse model [124].

A different study was designed to determination of the probiotic effect of *Lactobacillus fermentum* 296 on cardiometabolic disorders induced by high-fat diet in a rat model [122]. The probiotic was administrated by oral gavage to rats for 4 weeks. Results of this research suggest the ability of *Lactobacillus fermentum* 296 improve cardiovascular and biochemical parameters altered in cardiometabolic disorders. The increased production of short-chain fatty acids which modulate vasodilatation and induce hypotension has been proposed as possible mechanism of action this probiotic strain [122]. 

### 3.5. Autism Spectrum Disorders (ASD)

ASD is a collection of neurodevelopmental disorders (including impaired social interaction, communication, and repetitive and stereotyped patterns of behaviors) with evidence of genetic predisposition [125]. Intestinal imbalance and compositional changes in gut microbiome in ASD patients are reported. However, the role of intestinal microbiome in brain disorders is poorly documented. Children with ASD have often been reported to have gastrointestinal problems that are more frequent and more severe than in children from the general population. A strong correlation of gastrointestinal symptoms with the severity of ASD was confirmed in studies of the feces 58 children with ASD (of which half received a daily probiotic and half did not receive one) and 39 healthy children (who did not receive a probiotic) [107]. In the feces of children with ASD, significantly lower levels of SCFAs (including acetate, propionate and valerate) compared to healthy children and children with ASD taking probiotics were found. In addition, results of these research indicate an imbalance of gut microbiota in children with ASD (Table 5) [107].

### 3.6. Atopic Dermatitis (AD)

Presumably, intestinal microbiome dysbiosis is also associated with AD. This was confirmed in studies in which 19 children with atopic dermatitis and 18 healthy individuals were given a probiotic (*Bifidobacterium breve* BR03, *Lactobacillus salivarius* LS01) for 20 days. It was found that AD is characterized by dysbiosis of the intestinal microbiome with a predominance of some species such as *Faecalibacterium*, *Oscillospira*, *Bacteroides*, *Parabacteroides*, and *Sutterella*, that can act as possible biomarkers associated with the disease. In addition, a reduction or absence of some microorganisms, including those producing SCFAs (*Bifidobacterium*, *Blautia*, *Coprococcus*, *Eubacterium*, and *Propionibacterium*) with anti-inflammatory effects or involved in immune homeostasis, which might have a protective role against AD, has been identified. However, no significant changes were observed in the composition of intestinal microorganisms and the concentration of SCFAs in children with AD as a result of probiotic supplementation (Table 5) [108]. Therefore, differences in the composition of the intestinal microbiome of healthy children and those with AD may suggest that other probiotics be tested to restore intestinal homeostasis.

### 3.7. Gastrointestinal Disorders

SCFAs have a significant impact on preventing colonization of intestinal epithelium by pathogens. Current research shows that the effect of probiotic microorganisms in preventing pathogen infection is maintaining intestinal barrier or immune regulation [126]. Research on probiotics in gastrointestinal disorders has made huge progress in recent years. According to scientists *Lactobacillus casei* has anti-inflammatory effects on human intestinal epithelial cells infected by *Shigella* [127]. In addition, probiotic formulas can treat and prevent diarrhea caused by bacterial and viral infections [128]. Probiotics are effective in preventing *Clostridium difficile* infection [129]. Twenty-two children with shigellosis and 11 children with salmonellosis were examined for ten days, *Lactobacillus rhamnosus* GG and comparatively antibacterial drug (TMP-SMX or Polymyxin) were administered for five days and then the SCFAs content in the feces was evaluated. Iso-caproic acid was not found in children receiving the probiotic strain, but an increase in fecal propionic acid concentration was observed. Iso-caproic acid is not found in healthy adults and may be indicative of *Clostridium difficile* (Table 5) [106]. Probiotics prevented *C. difficile* infection [67] and in randomized clinical studies, prophylactic supplementation of *Lactobacillus* GG (LGG) to children reduced the incidence of HRV disease [68].

## 4. Conclusions

Maintaining the balance of the intestinal microbiome is crucial for maintaining normal human health and preventing many diseases. Short-chain fatty acids, as metabolites of intestinal bacteria, perform many important functions. The concentration of SCFAs depends on the composition and size of the population of intestinal microorganisms, genetic factors, environmental factors and the diet conditioning access to appropriate substrates. Imbalance of the intestinal microbiome and a decrease in the number of bacteria producing metabolites such as SCFAs (e.g., acetic, propionic and butyric acid) are often diagnosed in patients with inflammatory bowel diseases (IBD), irritable bowel syndrome (IBS), type 2 diabetes (T2D), obesity, infections bacterial, autoimmune disorders, or cancer patients. Numerous scientific reports confirm the effectiveness of probiotics in modulating the intestinal microbiome and their effect on the SCFAs content in the colon. Many studies, in addition to acting in the digestive system, concern the effect of SCFAs produced by intestinal microbiome on functions of distant tissues and organs. Researchers highlight the immunomodulatory effect of SCFAs produced by probiotics, but the mechanisms of their action still need further study. The use of probiotic microorganisms to prevent and treat intestinal dysbiosis, leading to an increase in SCFAs in the colon, seems to be an important direction for further research. Research on SCFAs in relation to diseases covered by the review is much-needed to understand their etiology and pathogenesis, and to propose new therapies. Clinical studies in human populations in this area are highly desirable.

## Figures and Tables

**Table 1:** Chemical and structural formulas of short-chain fatty acids (SCFAs) [29].

Name	Chemical Formula	Structural Formula	Molar Mass [g/mol]
Formic acid	HCOOH	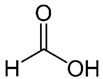	46.03
Acetic acid	CH_3_COOH	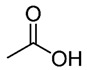	60.05
Propionic acid	CH_3_CH_2_COOH	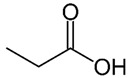	74.08
Butyric acid	CH_3_(CH_2_)_2_COOH	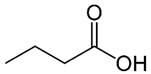	88.11
Valeric acid	CH_3_(CH_2_)_3_COOH	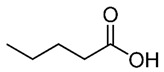	102.13
Caproic acid	CH_3_(CH_2_)_4_COOH	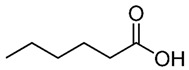	116.16

**Table 2:** Examples of commensal and probiotic microorganisms producing SCFAs and lactic acid [5].

Microorganism/s	Type	Acid/s	References
*Bifidobacterium* spp., *Blautia hydrogentrophica*, *Prevotella* spp., *Streptococcus* spp.	commensal	acetic	[33]
*Akkermansia muciniphilia*, *Bacteroides* spp.,	commensal	acetic, propionic	[33,34]
*Dalister succinatiphilus*, *Eubacterium* spp. (e.g., *E. halli*), *Megasphaera elsdenii*, *Phascolarctobacterium succinatutens*, *Roseburia* spp., *Salmonella* spp., *Veillonella* spp.	commensal	propionic	[34]
*Coprococcus* spp. (e.g., *Coprococcus catus*), *Roseburia inulinivorans*	commensal	propionic, butyric	[34,35,36]
*Anaerostipes* spp., *Coprococcus comes*, *Coprococcus eutactus*, *Clostridium symbiosum*, *Eubacterium rectale*, *Eubacterium hallii*, *Faecalibacterium* spp. (e.g., *Faecalibacterium prausnitzii*), *Roseburia* spp. (e.g., *Roseburia intestinalis*)	commensal	butyric	[33,34,35,36]
*Clostridium* spp., *Ruminococcus* spp.	commensal	acetic, propionic, butyric	[33,34,36,37]
*Bifidobacterium* spp.	probiotic	acetic, lactic	[38]
*Lactobacillus rhamnosus* GG (LGG), *Lactobacillus gasseri* PA 16/8	probiotic	propionic, lactic	[5]
*Bifidobacterium longum* SP 07/3, *Bifidobacterium bifidum* MF 20/5	probiotic	acetic, propionic, lactic	
*Lactobacillus salivarius spp salcinius* JCM 1230, *Lactobacillus agilis* JCM 1048	probiotic	propionic, butyric, lactic	[39]
*Lactobacillus acidophilus* CRL 1014	probiotic	acetic, propionic, butyric, lactic	[40,41,42,43]

**Table 3:** The characteristics of SCFAs and lactic acid receptors [33,59,60,61].

Receptor	Ligand	Protein G	Exspression	Physiological Function
**FFAR2—Free fatty acid receptor 2 (GPR43)**	Acetate, propionate, butyrate	Gi/o, Gq11	Small intestinal epithelium, colonic, colonic LP cells, leukocytes in small intestinal LP, adipocytes, polymorphonuclear cells, skeltal muscle, spleen and heart etc.	Apetite control, anti-lipolysis, increased insulin sensitivity, preadipocyte differentiation, expansion and differentiation of Tregs, protection against IBD, apoptosis of human colon cancer cel line etc.
**FFAR3—Free fatty acid receptor 3 (GPR41)**	Acetate, propionate, butyrate	Gi/o	Small intestinal epithelium, colonic, colonic LP cells (mast cells), peripheral nervous system, peripheral mononuclear cells, bone marrow spleen, adipocytes, lymph nodes, etc.	Leptin expression, oxygen consumption rate, increased energy expenditure, decreased food intake, hematopoiesis of DCs from bone marrow, increased DC precursors alleviating asthma and Treg cells etc.
**HCA1—Hydroxycarboxylic acid receptor 1 (GPR81)**	lactate	(Gi)	Predominantly in adipose tissue, minor in kidney, skeletal muscle, liver, intestinal tissue, rat and human brain, mouse primary cortical neuronal cells, macrophages, etc.	Modulation of cortical neuron activity, and enterocyte turnover in response to starvation-refeeding, anti-lipolysis, anti-inflammatory on macrophages, reduced symptom of cancer and IBD in mouse models of hepatitis and pancreatitis, etc.
**HCA2—Hydroxycarboxylic acid receptor 2 (GPR109A)**	Niacin, ketone bodies, β-hydroxybutyric acids, butyrate	Gi/o, G*βγ*	Apical membrane of colonic and small intestinal epithelium, monocytes, adipocytes, macrophages, DCs, neutrophils, retinal pigment epithelium, etc.	Improved epithelial barrier function, anti-lipolysis, decrease of triglyceride, protection against CRC and colitis, increase of Treg generation and IL-10 producing T cells, etc.
**Olfr78 (murine) OR51E2 (human)**	Acetate, propionate	NR	Neurons, epithelial enteroendocrine cells of colon, enteroendocrine cells, renal afferent arteriole, smooth muscle cells, etc.	Regulation of hormone secretion (GLP-1, PYY) and blood pressure, etc.
**PPARγ (Peroxisome proliferator-activated receptor gamma)**	Propionate, butyrate	NR	Large intestine adenocarcinoma cells, etc.	Regulation of lipid metabolism, a joining factor between the gut microflora composition and accumulation of the adipose tissue, etc.

Abbreviations: CRC—colorectal cancer; DC—dendritic cell; GLP-1, glucagon-like peptide; GPR—G-protein coupled receptor; IBD—inflammatory bowel disease; IL-10 (interleukin-10); LP—lamina propria; NR—not reported; Olfr—olfactory receptor; PYY—peptide YY; Treg—regulatory T cell.

**Table 4:** Examples of trials regarding the effect of SCFAs on human health.

Type of SCFA	The Effect on Human Health	References
**Acetate**	Protection against *E. coli* O157:H7 infection	[82]
	Participates in the synthesis of cholesterol	[74]
**Butyrate**	Is the source of 70% of the energy used by intestinal epithelial cells	[37]
	Increases in MUC2 gene expression and the production of mucin	[64]
	inhibits development of tumor cells and inducing the process of their apoptosis	[65,66,67]
	Inhibits the genotoxic activity of nitrosamides and hydrogen peroxide	[83]
	Has immunoregulatory effect	[78]
	Plays a role in the prevention and the treatment of distal ulcerative colitis, Crohn’s disease and cancer	[84]
	Improves ulcerative colitis (UC) symptoms	[85]
**Butyrate/acetate/propionate**	Improves the macroscopic and histological signs of inflammation	[86]
**Formate**	Presence is associated with methanogenesis and its concentration may be elevated during inflammation	[71,72]
**Propionate**	Decreases cholesterol synthesis in the liver, improves lipid metabolism	[76,87]
	Has anti-proliferative effect	[88,89]
**Valerate**	Stimulates the growth of intestinal epithelium Has a beneficial effect on the pathogenesis of diseases such as colitis, cardio-metabolic diseases and cancer	[79,80,81]

**Table 5:** Examples of clinical trials regarding the effect of probiotics on SCFAs production by human intestinal microbiota.

Subjects	Probiotic	Time of Administration	Main Outcome	Ref.
**Colorectal Cancer (CRC)**				
30 patients (10 CRC patients and 20 healthy subjects)	*Lactobacillus gasseri* OLL271 6: LG21	12 weeks	↑ number of *Lactobacillus* spp. ↓ number of *Clostridium perfringens* in intestinal population; ↑ concentration isobutyric acid in feces and natural killer (NK) cell activity; ↓ pH and the synthesis of fecal putrefaction products.	[103]
17 healthy subjects (aged 45 to 75 years)	*Bifidobacterium lactis LAFTI B94*	4 weeks	↑ number of *Bifidobacterium lactis* in intestinal population; no changes in the pH, the SFCA fecal concentration, the serum hs-CRP and cytokines and also the crypt proliferation and cell height.	[104]
**Obesity**				
40 children 7–10 years (19 normal weight and 21overweight children)	*Lactobacillus casei* Shirota	2 phases (each lasted for 4 weeks with a 4-week wash-out period between phases)	↑ number of *Lactobacillus* spp. and *Bifidobacterium* spp.; ↑ the total SCFAs and propionic acid contents in normal weight and overweight children.	[2]
34 children 8.5–10.8 years (22 normal weight and 12 overweight children)	*Lactobacillus casei* Shirota	6 months	↓ weight; improving the lipid metabolism in children with obesity; ↑ number of *Bifidobacterium* spp. and the acetic acid concentration in the feces.	[24]
**Type 2 Diabetes**				
50 volunteers with T2D	*Lactobacillus acidophilus* La-5, *Bifidobacterium animalis* subsp. *lactis* BB-12	6 weeks	the proportion of C3:C2:C4 acids, taking into account the mean values, was also similar: 10:8:1 in the control group and 14:10:1 in the probiotic group, improving glycemic control.	[105]
**Gastrointestinal Disorders**				
22 children with shigellosis and 11 children with salmonellosis (mean age–5.3 years)	*Lactobacillus rhamnosus* GG (ATCC 53103)	In three portions per day for 10 days compared to treatment with an antibacterial drug (TMP-SMX or Polymyxin) for 5 days.	acetic, propionic and iso-valeric acid were significantly higher in shigellosis than in salmonellosis. ↑ concentration of propionic acid by the 5th day of treatment; difference in iso-caproic acid in the 10th day samples: it was not found in any child who had received probiotic but was present in half of the samples from the group treated solely with antibacterial drug.	[106]
**Autism Spectrum Disorders**				
97 children (58 children with ASD–two groups: A-Probiotic, A-No-Probiotic and 39 healthy children) (2.5–18 years)	No information		↓ level of acetate, propionate and valerate and total SCFAs in children with autism; the imbalance of gut microbiota in children with autism.	[107]
**Atopic Dermatitis**				
19 AD children and 18 healthy individuals (0–6 years)	*Bifidobacterium breve* BR03, *Lactobacillus salivarius* LS01	20 days	an alteration in AD microbiome composition with the depletion or absence of some species; ↓ SCFAs producing bacteria.	[108]
